# Glucosamine Modified the Surface of pH-Responsive Poly(2-(diethylamino)ethyl Methacrylate) Brushes Grafted on Hollow Mesoporous Silica Nanoparticles as Smart Nanocarrier

**DOI:** 10.3390/polym12112749

**Published:** 2020-11-20

**Authors:** Abeer Beagan, Shatha Lahmadi, Ahlam Alghamdi, Majed Halwani, Mohammed Almeataq, Abdulaziz Alhazaa, Khalid Alotaibi, Abdullah Alswieleh

**Affiliations:** 1 Department of Chemistry, College of Science, King Saud University, Riyadh 11451, Saudi Arabia; 438204365@student.ksu.edu.sa (S.L.); 437204374@student.ksu.edu.sa (A.A.); khalid.m@ksu.edu.sa (K.A.); 2 Nanomedicine Department, King Abdullah International Medical Research Center, King Saud bin Abdulaziz University for Health Sciences, Riyadh 11481, Saudi Arabia; halawanima@NGHA.MED.SA; 3 King Abdulaziz City for Science and Technology, Riyadh 11442, Saudi Arabia; mmeataq@kacst.edu.sa; 4 Department of Physics and Astronomy, College of Science, King Saud University, Riyadh 11451, Saudi Arabia; aalhazaa@ksu.edu.sa

**Keywords:** hollow mesoporous silica nanoparticles, pH-Responsive polymer brushes, surface modification, drug delivery nanosystem, anticancer drug

## Abstract

This work presents the synthesis of pH-responsive poly(2-(diethylamino) ethyl methacrylate) (PDEAEMA) brushes anchored on hollow mesoporous silica nanoparticles (HMSN-PDEAEMA) via a surface-initiated ARGET ATRP technique. The average size of HMSNs was ca. 340 nm, with a 90 nm mesoporous silica shell. The dry thickness of grafted PDEAEMA brushes was estimated to be ca 30 nm, as estimated by SEM and TEM. The halogen group on the surface of PDEAMA brushes was successfully derivatized with glucosamine, as confirmed by XPS. The effect of pH on the size of the hybrid nanoparticles was investigated by DLS. The size of fabricated nanoparticle decreased from ca. 950 nm in acidic media to ca. 500 nm in basic media due to the deprotonation of tertiary amine in the PDEAEMA. The PDEAEMA modified HMSNs nanocarrier was efficiently loaded with doxorubicin (DOX) with a loading capacity of ca. 64%. DOX was released in a relatively controlled pH-triggered manner from hybrid nanoparticles. The cytotoxicity studies demonstrated that DOX@HMSN-PDEAEMA-Glucosamine showed a strong ability to kill breast cancer cells (MCF-7 and MCF-7/ADR) at low drug concentrations, in comparison to free DOX.

## 1. Introduction

Nanoparticles have been widely used as drug delivery systems (DDSs), specifically for anticancer therapy. The size of the nanoparticles is 100- to 10,000-fold smaller than that of cancer cells; therefore, they are able to cross the cell barriers easily [1]. Among the various nanoparticles that have been utilized as anticancer nanocarriers, including liposomes [2], polymeric nanoparticles [3], nucleic acid [4], carbon [5], and silica nanoparticles [6,7], the latter stand out for the manufacture of DDSs due to their high surface area, high rigidity, thermal stability, biocompatibility, high loading and protection of the drug, controllable rate of release, and efficient targeting [8,9,10,11,12,13,14]. Thus, different types of silica nanoparticles such as mesoporous silica nanoparticles (MSNs) and hollow mesoporous silica nanoparticles (HMSNs) have been used as nanocarriers [15].

HMSNs have a central cavity that is beneficial for the loading of drugs. Previous studies have shown that HMSNs can store a higher amount of drugs than conventional MSNs [16,17,18]. Zhu et al. used ibuprofen to examine the storage capacity of HMSNs and MSNs. They found that 744.5 and 358.6 mg/g of ibuprofen molecules can be stored in HMSNs and MSNs, respectively [17]. Geng et al. prepared MSNs and HMSNs as drug carriers for carvedilol and fenofibrate using solvent evaporation and adsorption equilibrium methods to investigate the different drug loading ability of HMSNs and MSNs [18]. Their results showed that HMSNs had higher drug loading than MSNs in both methods. However, the control of drug release is more limited for HMSNs than for MSNs. To achieve better control of the drug release, the HMSN surface can be modified with organic molecules such as stimuli-responsive polymer brushes [19,20].

Polymer brushes can be anchored by the end of the polymer chains to the surface of particles via physisorption or covalent attachment [20,21]. Interestingly, some of these polymer brushes are stimuli-responsive; environmental changes such as light, temperature, and pH can alter the polymer chain conformation [22,23,24]. Various techniques have been used to grow polymer brushes on nanoparticle surfaces, including reversible addition–fragmentation chain transfer polymerization (RAFT) and atom transfer radical polymerization (ATRP) [25,26,27].

To achieve a controlled drug release in specific tumor sites, mesoporous silica has been modified with pH-responsive polymer brushes, affording a gatekeeper to open the pores in either lower pH or higher temperature conditions [28,29,30]. Nam-Kyoung et al. reported the synthesis of a novel pH-triggered drug delivery system by grafting poly-L-lysine on the pore entrances of MSNs as a drug gatekeeper [31]. In addition, the synthesis of a pH-responsive diblock copolymer, i.e., 2-(*tert*-butylamino)ethyl methacrylate-*b*-poly(ethylene glycol) methyl ether methacrylate, was reported by Alswieleh et al. [32]. Doxycycline was loaded in the multifunctional pH-responsive diblock copolymer, and the drug could be released from the nanosystem in a relatively controlled manner. Yang et al. designed a pH and glutathione dually responsive HMSNs-coated poly(acrylic acid) shell for controlled drug delivery [33]. The loading capacity and encapsulation efficiency were found to be 43% and 96%, respectively. Yu et al. reported the growth of poly(*N*,*N*-dimethylaminoethyl methacrylate) on the HMSN surface by surface-initiated (SI)-ATRP [34]. The results showed that the hybrid nanomaterials have a large storage capacity with controlled release behavior. Zhang et al. modified HMSNs with poly(2-(diethylamino)ethyl methacrylate) (PDEAEMA) via SI-ATRP, [35] finding that the drug was easily encapsulated into and quickly released from the nanosystem with a high loading capacity. Another HMSN system coated with a copolymer shell bearing *N*-(3,4-dihydroxyphenethyl) methacrylamide and *N*-isopropylacrylamide reported by Zhang et al. exhibited a high drug loading capacity and embedding efficiency [36].

Despite all of these advances, to the best of our knowledge, very little work has been done on the modification of the polymer brush surface. Herein, HMSNs were synthesized with an average particle size of 340 nm. The as-prepared HMSNs were coated with pH-responsive poly(2-(diethylamino) ethyl methacrylate) (PDEAEMA) brushes via SI-activators regenerated by the electron transfer (ARGET) ATRP method. The halogen group on the surface of the PDEAMA brushes was then converted to amine, followed by the reaction with succinic anhydride and derivatization with glucosamine. The effect of pH on the size of the fabricated nanoparticles was investigated by dynamic light scattering (DLS). The nanocarriers were loaded with doxorubicin (DOX) in acidic media. The DOX release behavior was studied at different pH values. The nanosystem was characterized using various techniques such as scanning electron microscopy (SEM), transmission electron microscopy (TEM), and X-ray photoelectron spectroscopy (XPS). The cytotoxicity studies were performed using breast cancer cells (MCF-7 and MCF-7/ADR) at low drug concentrations.

## 2. Materials and Methods

### 2.1. Materials

Deionized water (DI) was obtained from the Elga Pure Nanopore System. *N*-Cetyltrimethylammonium bromide (CTAB, 98%), ammonium hydroxide (28 wt%), tetraethylorthosilicate (TEOS, 98%), 3-aminopropyltriethoxysilane (APTES, >98%), methanol (99.8%, HPLC grade), ethanol (99.8%, HPLC grade), dimethylformamide (99.9%, HPLC grade), pyridine (analytical grade), dichloromethane (DCM, HPLC grade), 2,2′-bipyridyl (BIPY, 99%), 2-bromo-2-methylpropionylbromide (BIBB, 98%), PDEAEMA (99%), and triphenylphosphine (PPh_3_, 99%) were purchased from Sigma-Aldrich (Taufkirchen, Germany). Cupric bromide (CuBr_2,_ 98%) and cuprous chloride (CuCl, 98%) were obtained from BDH (British Drug Houses, London, UK). Sodium azide (NaN_3,_ 99%) and triethylamine (TEA, 99%) were purchased from Loba Chemie (Ditzingen, Germany). Tetrahydrofuran (THF) was purchased from Nexgen Chemicals (Maharashtra, India). Ascorbic acid (98%) was obtained from Riedel-de Haën. Ammonium nitrate (NH_4_NO_3_, 99%) was obtained from Winlab (Harborough, UK). Succinic anhydride, *N*-(3-dimethylaminopropyl)-*N*′-ethylcarbodiimide hydrochloride (EDC), *N*-hydroxysuccinimide (NHS), and doxorubicin hydrochloride (DOX·HCl) were obtained from Tokyo Chemical Industry (Japan). Sodium carbonate, sodium chloride, potassium chloride, disodium hydrogen phosphate, and potassium dihydrogen phosphate were obtained from Alfa Aesar (Lancashire, UK). All the chemicals were used as received.

### 2.2. Methods

#### 2.2.1. Synthesis of Uniform HMSNs

HMSNs were prepared according to the method published by Fang et al. [37] The SiO_2_ core was firstly synthesized via a modified Stöber method. Briefly, ethanol (74 mL), 10 mL of DI water, and ammonium aqueous solution (28%, 3.15 mL) were mixed; then, 6 mL of TEOS was added, and the mixture was stirred at room temperature for 1 h. The SiO_2_ product was collected by centrifugation at 6000 rpm for 15 min and washed with DI water and ethanol.

Secondly, the as-prepared SiO_2_ particles were coated with a mesoporous shell (SiO_2_@MSN) as follows: SiO_2_ (50 mg) was homogeneously dispersed in 10 mL of DI water by ultrasonication for 15 min. The SiO_2_ suspension was mixed with a solution containing CTAB (75 mg), DI water (15 mL), ethanol (15 mL), and ammonia solution (0.275 mL) under stirring at room temperature for 45 min. Then, 0.125 mL of TEOS was added to the mixture and allowed to react for 3 h. The SiO_2_@MSN nanoparticles were collected and washed with DI water and ethanol.

Thirdly, HMSNs were prepared by etching the SiO_2_ in SiO_2_@MSN to form a hollow core. Thus, SiO_2_@MSN was dispersed in 10 mL DI and sonicated for 15 min. Na_2_CO_3_ (212 mg) was added to the mixture and stirred at 50 °C for 15 h. The final product was centrifuged and washed with DI water and ethanol.

#### 2.2.2. Synthesis of HMSNs Modified with Amine Groups (HMSN-NH_2_)

To functionalize the HMSN surface with amine groups, 1.5 g of HMSNs was dispersed into methanol (50 mL) containing 1.5 mL of APTES, and the mixture was refluxed for 12 h. The obtained solution was centrifuged and washed with ethanol several times to remove the residual APTES.

#### 2.2.3. Formation of Channels of HMSNs

The surfactant (CTAB) was removed by suspending 1 g of HMSN-NH_2_ in a solution of ammonium nitrate/ethanol (1 g/100 mL) under reflux and stirring overnight. The final product was obtained by centrifugation, washed with ethanol three times, and dried under a vacuum overnight.

#### 2.2.4. Immobilization of BIBB Initiator on HMSNs (HMSN-Br)

HMSN-NH_2_ (1 g) was dispersed in DCM (25.0 mL) and TEA (0.5 mL, 3.6 mmol). Then, 0.25 mL (2.02 mmol) of 2-bromo-2-methylpropionyl bromide was mixed with 5 mL of DCM, added dropwise into the suspension, and allowed to react for 48 h at room temperature. The HMSN-Br nanoparticles were collected by centrifugation and washed with DCM and ethanol [38].

#### 2.2.5. Synthesis of Poly(2-(diethylamino)ethyl Methacrylate) Brushes-Grafted HMSNs (HMSN-PDEAEMA)

In a typical reaction, 0.4 g of HMSN-Br, 12 mL of ethanol, and 3 mL of water were mixed and degassed with N_2_ for 30 min under stirring. 2-Diethylaminoethyl methacrylate (2 mL), 0.0009 g of CuBr_2_, and 0.0067 g of BIPY were added to the mixture. Ascorbic acid (0.0076 g) was introduced into the mixture under N_2_ and stirred at room temperature for 6 h. The products were collected by centrifugation and washed with acidic aqueous solution to remove any copper residue, followed by washing with ethanol.

#### 2.2.6. Surface Coating of HMSNs with Amine Groups (HMSN-PDEAEMA-NH_2_)

HMSN-PDEAEMA (200 mg) was dispersed in 2 mL of degassed DMF and kept under N_2_. In a separate flask, NaN_3_ was degassed, then DMF was added to give a 0.2 M solution. The saturated solution of NaN_3_ (5 mL) was then added to HMSN-PDEAEMA under N_2_, and the resulting mixture was heated at 60 °C for 18 h. Afterwards, the particles were separated by centrifugation and washed three times with DMF.

The obtained product was suspended in 2 mL of degassed DMF and kept under N_2_. In a separate flask, PPh_3_ was degassed, and then DMF was added to give a 0.2 M solution. The saturated solution of PPh_3_ (5 mL) was added to HMSN-PDEAEMA under N_2_, and the mixture was heated at 60 °C for 18 h. Then, the particles were separated by centrifugation and washed with DMF, ethanol, and water. The sample was mixed with water/THF (5/5) under N_2_ and stirred for 18 h at 40 °C. The final product was obtained by centrifugation and washed with ethanol.

#### 2.2.7. Immobilization of Glucosamine on the Surface of HMSNs (HMSN-PDEAEMA-Glucosamine)

HMSN-PDEAEMA-COOH was obtained by suspending 100 mg of HMSN-PDEAEMA-NH_2_ in a solution of DCM:pyridine (1:1) and sonicating for 10 min. Succinic anhydride (500 mg) was added to the reaction mixture and sonicated for 30 min. The resulting mixture was stirred at room temperature for 18 h. The sample was separated by centrifugation and washed with DMF to remove excess succinic anhydride.

The product (100 mg) was suspended in 2 mL of DMF and sonicated for 10 min. EDC (200 mg) and NHS (200 mg) were added to the suspension and sonicated for 30 min. The resulting mixture was stirred at room temperature for 18 h to afford HMSN-PDEAEMA-NHS. The sample was separated by centrifugation and washed with DMF to remove excess EDC and NHS [39].

**Scheme 1:**
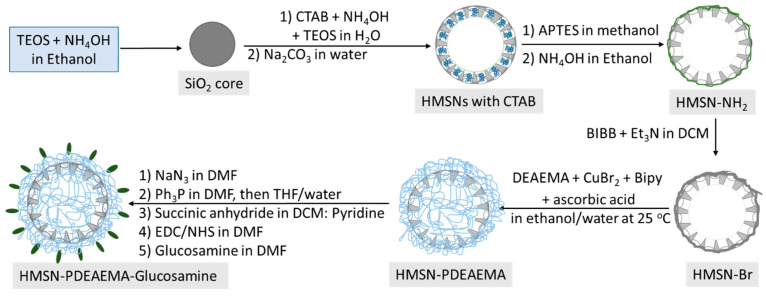
Illustration of the synthesis route of glucosamine-modified pH-responsive poly(2-(diethylamino)ethyl methacrylate) brushes grafted on hollow mesoporous silica nanoparticles.

HMSN-PDEAEMA-NHS (100 mg) was suspended in 10 mL of DMF and sonicated for 30 min. Glucosamine (0.7 mg) and TEA (100 µL) were added to the suspension at room temperature for 24 h. The final product was separated by centrifugation and washed with DMF and DI water to remove unreacted glucosamine (Scheme 1).

### 2.3. Measurement and Characterization

The nanoparticles were imaged by SEM using a JEOL JSM-7610F microscope (Japan) at 15 kV without any pretreatment. For TEM imaging, a JEOL JEM-1400 microscope was used at 100 kV by placing a drop of nanoparticles diluted in ethanol on a copper grid and dried at 60 °C. Infrared (IR) spectra were acquired using a Perkin-Elmer Spectrum BX instrument (USA), using KBr pellets at the region of 400–4400 cm^−1^ with a resolution of 4 cm^−1^. The surface area, pore volume, and pore size were measured using N_2_ physisorption isotherms on a Micrometrics Gemini 2375 volumetric analyzer (USA). Before analysis, samples were degassed at 140 °C for 10 h. Thermogravimetric analysis (TGA) was performed on a Perkin-Elmer Pyris 1 TGA instrument (USA) in a temperature range of 25–600 °C and a heating rate of 20 °C/min. XPS measurements were used (model number JPS-9030) manufactured by JOEL company, Japan. All samples were etched for 20 s by Ar gas to remove surface contamination inside an Ultra High Vacuum Chamber (UHV) of about 10^−9^ Torr. Particle size was measured at different pH values using DLS Malvern instruments (Zetasizer Nano ZS, UK) at 25 °C. UV spectra were obtained using a SpectraMax Plus 384 microplate reader (USA).

### 2.4. Drug Loading and Release

The procedure of drug loading and release was conducted according to the method published by Bilalis et al. [40]. Accordingly, the fabricated nanoparticles (1 mg) were suspended in 1 mL of PBS; then, 1 mL of DOX solution (2 mg/mL) was added to the suspension. The pH of the suspension was adjusted to 3 by adding an aqueous solution of HCl (0.1 M) and stirred for 24 h at room temperature in the dark condition. The pH of the suspension was changed to 8 by adding an aqueous solution of NaOH (0.1 M) and stirred for 2 h. The supernatants were collected to determine the concentration of unloaded DOX using an UV–vis spectrophotometer at 480 nm. The entrapment efficiency (EE) and loading capacity (LC) for DOX were calculated by the following formulas, respectively:

EE = (weight of drug in nanoparticles/weight of drug added initially) × 100(1)

LC = [weight of DOX/(weight of nanoparticles + weight of DOX)] × 100(2)

In the release procedure, 0.25 mg of DOX@HMSN-PDEAEMA-glucosamine or DOX@HMSN-PDEAEMA was added into 1 mL of PBS with different pH values (5, 6.5, 7.4, 8) at 37 °C under constant shaking (220 rpm). The solution was collected from the dispersion at different time periods, and the amount of released drug was determined by UV–vis spectroscopy. The volume of buffer was kept constant by adding (1 mL) of fresh medium after each collection. The cumulative weight percent of DOX was calculated using the following equation:

Cumulative weight (%) = (weight of drug at specific time points/weight of drug in nanoparticles) × 100(3)

### 2.5. Cytotoxicity Assay

The cytotoxicity of the hybrid HMSNs was evaluated by using the viability reagent PrestoBlue. The PrestoBlue kit relies on resazurin substrate (water-soluble dye and non-toxic molecule), and it can be effectively reduced in mitochondria to resorufin by NADPH dehydrogenase or NADH dehydrogenase as a measurement for mitochondrial metabolic activity. This conversion occurs intracellularly, where resorufin-formed molecules enter the cytosol as indicators for cell death and viability [41]. Breast cancer cells lines (MCF-7 and MCF-7/ADR) were harvested using 0.25% trypsin-EDTA and seeded into 96-well plates at a density of 3000 cells/well in DMEM media and cultured in 5% CO_2_ at 37 °C for 24 h. On the second day, media were removed and replaced by media with different drug forms of Free-DOX, DOX@HMSN-PDEAEMA, and DOX@HMSNs-PDEAEMA-Glucosamine with concentrations of 2931, 1466, 733, 366.37, 183, 92, 46, 23, 11, 6, and 3 µM, and the cells plates were incubated in 5% CO_2_ at 37 °C for 24 h. Finally, plates were subjected to PrestoBlue kit procedures to measure cell viability as instructed in the kit manual.

### 2.6. Data Analysis

GraphPad-Prism software was used to plot the figures and to conduct the data analysis, and NOVA and Tukey’s multiple comparisons test analysis were used to compare groups and measure the *p*-value. A (*p* < 0.05) was considered significant. All experiments were done in triplicate.

## 3. Results and Discussion

**Figure 1:**
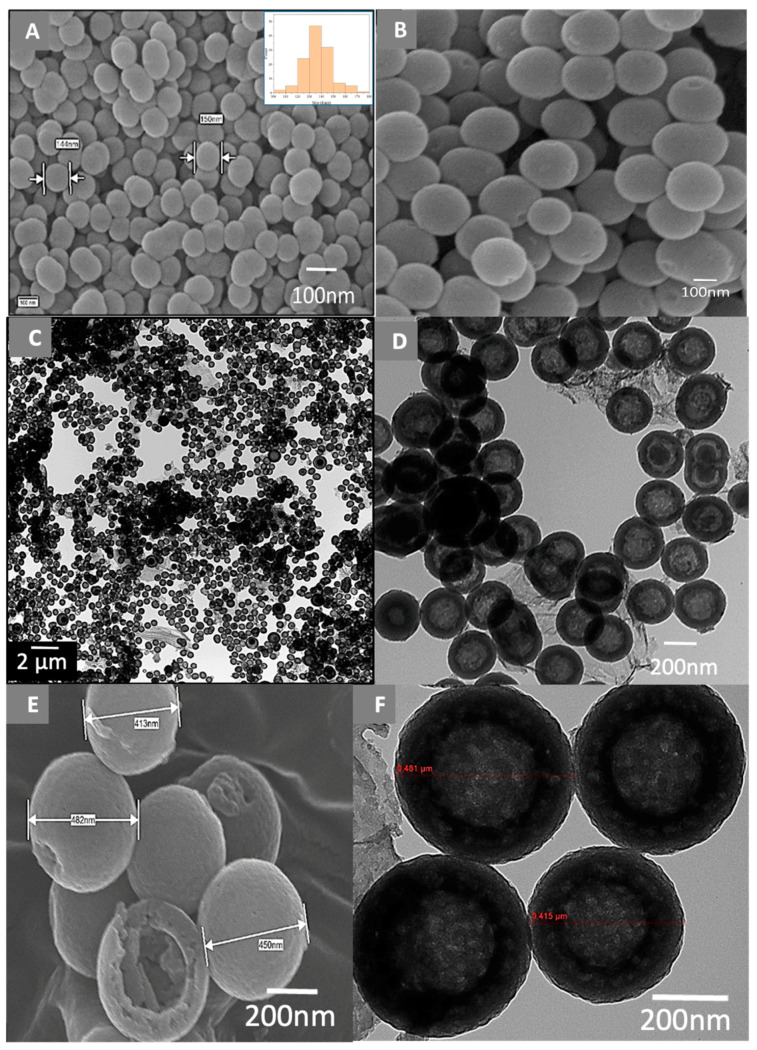
(**A**) SEM image of SiO_2_ core synthesis by Stober method; (**B**) SEM image of SiO_2_ core coated with mesoporous shell; (**C**,**D**) TEM images of hollow mesoporous silica nanoparticles after etching by Na_2_CO_3_ at 50 °C for 15 h; (**E**) the SEM image; (**F**) the TEM image of hollow mesoporous silica nanoparticles coated with PDEAEMA.

The silica cores were synthesized via the Stöber method in the presence of a silica source and an aqueous basic-alcohol medium. The obtained silica nanoparticles had a spherical shape, smooth surface, and a monodispersed diameter of around 140 nm, as shown in Figure 1A. The size distribution was estimated by image J software to be between 120 and 180 nm. The SiO_2_ nanoparticles were then coated with a silica shell in the presence of CTAB, leading to the formation of a mesoporous shell. The mean shell thickness was ca. 100 nm with a particle size distribution between 230 and 340 nm (Figure 1B). After etching by Na_2_CO_3_ for removal of the silica cores, hollow silica nanoparticles were formed, as can be seen in the TEM images depicted in Figure 1C,D. Most HMSNs were formed uniformly with a narrow size distribution. The average size of HMSNs was estimated to be ca. 340 nm, which agrees with the SEM image profile. The thickness of the shell was measured to be ca. 90 nm.

PDEAEMA brushes were successfully grafted on the surface of HMSNs via ATRP, as illustrated in the SEM and TEM images shown in Figure 1E,F. The average diameter of the spherical HMSN-PDEAEMA was larger than that of HMSNs (ca. 400 nm vs. 340 nm, respectively). The dry thickness of the PDEAEMA brushes was estimated to be 30 nm. The TEM image shows that the size of the nanoparticles was ca. 410 nm, which is in good agreement with the result obtained from the SEM image. The thickness of the silica shell and dry polymer was ca. 30 nm.

**Figure 2:**
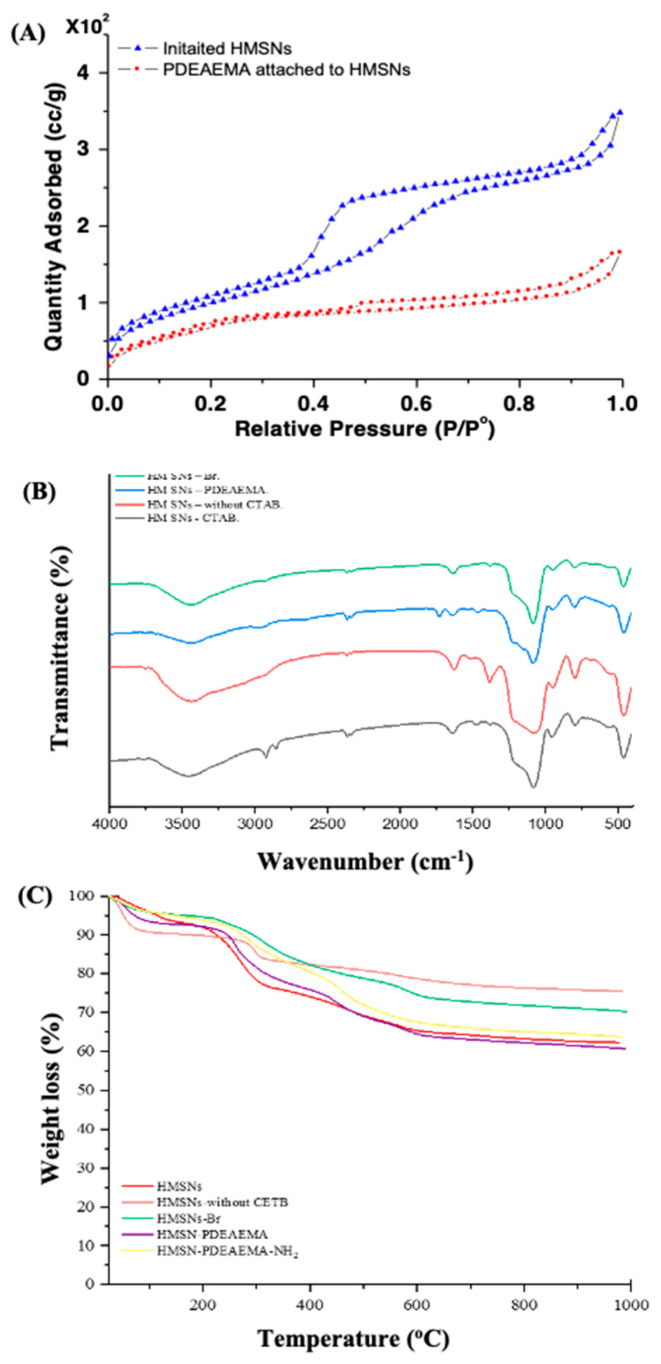
(**A**) Brunauer–Emmett–Teller measurement of HMSN-Br and HMSN-PDEAEMA; (**B**) FTIR spectra of HMSN-CTAB, HMSNs without CTAB, HMSN-PDEAEMA, and HMSN-Br; (**C**) thermogravimetric analysis curves of HMSNs, HMSNs without CTAB, HMSN-Br, HMSN-PDEAEMA, and HMSN-PDEAEMA-NH_2._

Figure 2A shows the N_2_ adsorption–desorption isotherms for HMSN-Br and HMSN-PDEAEMA, which exhibit a typical IV-type curve, indicating the presence of mesoporous materials. The surface area and pore volume of HMSN-Br were 372 m^2^·g^−1^ and 0.45 cm^3^·g^−1^, respectively. After grafting PDEAEMA, the resulting HMSN-PDEAEMA showed a decrease in the surface area (237 m^2^·g^−1^) and pore volume (0.18 cm^3^·g^−1^). These results demonstrate that PDEAEMA blocked the entrance of the mesoporous channels.

The FTIR spectra of HMSN-CTAB, HMSNs without CTAB, HMSN-Br, and HMSN-PDEAEMA are shown in Figure 2B. For all samples, the absorption peaks at 1086, 800.38, and 463.79 cm^−1^ can be attributed to Si–O–Si bonds, and the absorption peaks at 3450 and 958.79 cm^−1^ were assigned to the stretching absorption vibration of Si–OH. For HMSN-CTAB, C–H stretching bands were shown at 2926 and 2856 cm^−1^. These peaks disappeared after the extraction of the surfactant. A new absorption peak appeared in the spectra of HMSN-PDEAEMA at 1732 cm^−1^ as compared with HMSN-Br, indicating the presence of C=O groups. These results are indicative of the successful growth of the polymer brushes.

The successful modification of HMSNs was also evaluated by TGA by heating to 1000 °C in an N_2_ atmosphere (Figure 2C). As shown in the TGA curves, the increased weight loss after each synthetic step further confirmed the modification of the HMSN surface. It was found that the weight loss of HMSNs with CTAB exhibited three mass loss steps. The first step ranged from ambient temperature to about 220 °C and is attributable to water desorption and dehydration from the materials. The second step occurred from ca. 300 to 480 °C and is assigned to the degradation of CTAB. The third mass loss step, which was around 580 °C, can be attributed to the dehydroxylation of the materials. The weight loss of HMSNs with CTAB was about 38%. The TGA curve of the amino-functionalized HMSNs after removing CTAB showed four mass loss steps. The first step ranged from room temperature to ca. 100 °C and is ascribed to water desorption from the surface of the nanoparticles. The second step occurred from ca. 100 to 300 °C and is due to dehydration from the materials. The third mass loss step was around 350 to 480 °C and is attributable to the degradation of APTES. The fourth mass loss step, which occurred around 580 °C, can be assigned to dehydroxylation. The weight loss was ca. 20%. Similar behavior was observed when the polymerization was initiated, with a weight loss of 25%. After the polymerization, three mass loss steps were observed. The first step, ranging from ambient temperature to about 230 °C, is attributed to water desorption and dehydration from the materials. The second step occurred from ca. 300 °C to 450 °C, corresponding to polymer degradation. The third step is assigned to the degradation of the saline monolayer and CTAB at a temperature ranging from 480 to 550 °C. The fourth mass loss step was around 580 °C and is attributable to dehydroxylation of the materials. The weight of PDEAEMA attached to the HMSN surface was estimated to be ca. 20%.

**Figure 3:**
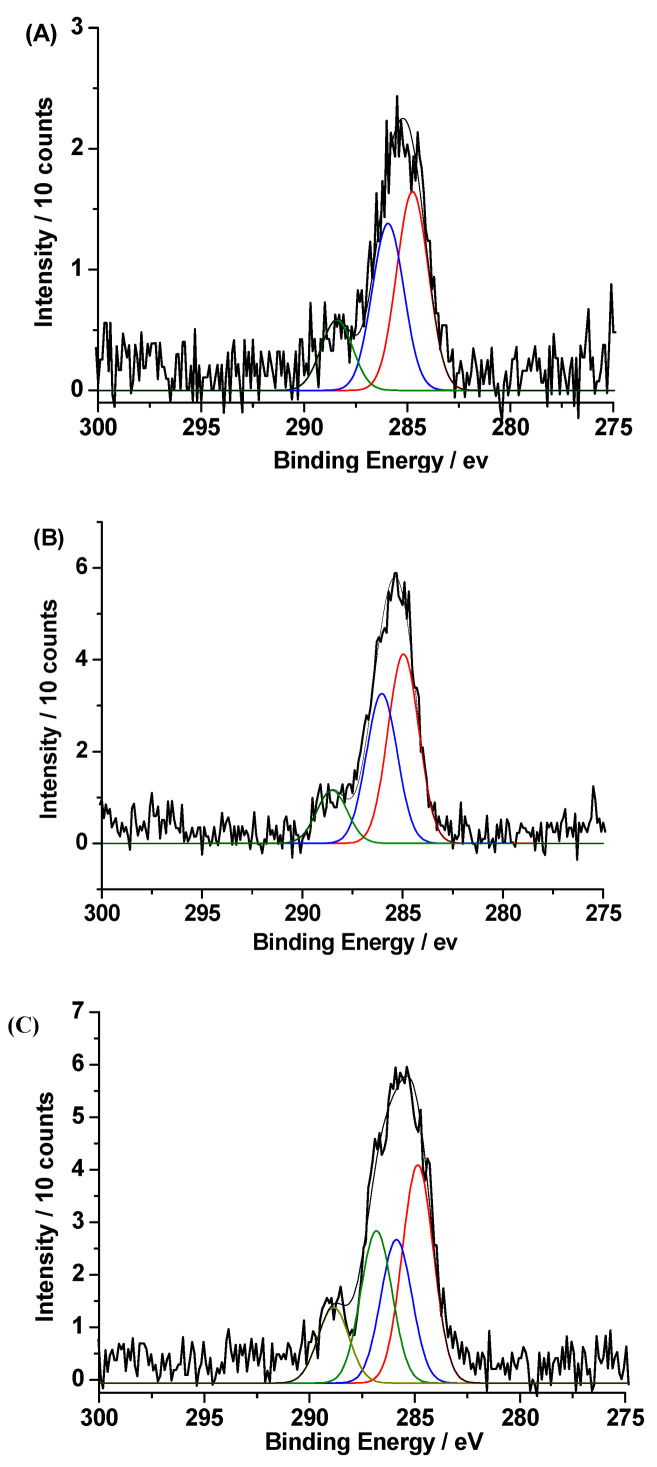
X-ray photoelectron spectra at the C1s region for: (**A**) HMSN-PDEAEMA; (**B**) HMSN-PDEAEMA-NH_2_; (**C**) HMSN-PDEAEMA-glucosamine.

XPS characterizations were conducted to determine the surface composition of HMSN-PDEAEMA before and after glucosamine modification (Figure 3). At the C1s region (Figure 3A), three peaks at binding energies of 284.9, 286, and 288.5 eV with a ratio of 4.8:3.9:1 correspond to the C–H, C–(N, O), and O=C–O bonds of PDEAEMA, respectively. The peak areas were similar to the theoretical ratio 5:4:1, which corresponds to the polymer composition. As expected, no noticeable difference was observed in the polymer peaks after debromination (Figure 3B). After the immobilization of glucosamine, a peak appeared at ~286.5 eV, which can be attributed to the increase in the surface content of C–O bonds from glucosamine. An increase in the intensity of the peak at 288.5 eV confirmed the successful attachment of glucosamine (Figure 3C).

**Figure 4:**
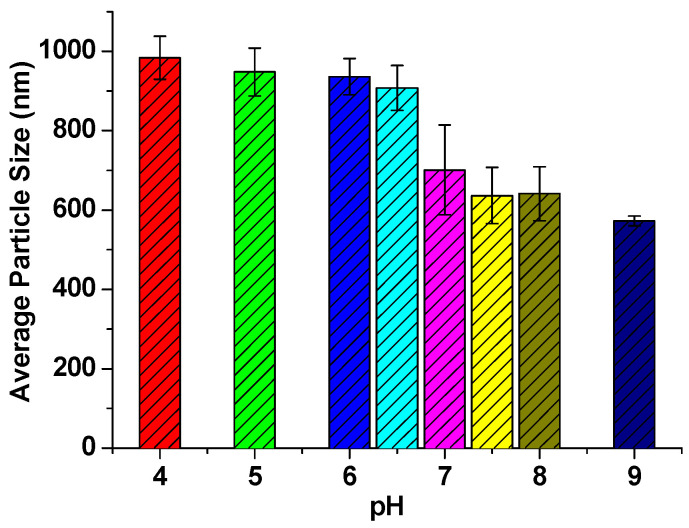
The average particle size of HMSN-PDEAEMA in PBS buffer solution with different pH values at room temperature.

As shown in Figure 4, the hydrodynamic sizes of HMSN-PDEAEMA dispersions in PBS at different pH values were measured by DLS. As expected, the size of HMSN-PDEAEMA was dependent on the pH value due to protonation or deprotonation of the tertiary amine groups at the side-chain of PDEAEMA, which affected the polymer polarity. At an acidic pH, the hydrodynamic size of HMSN-PDEAEMA increased due to the hydrophilic characteristic caused by protonation. The size of the particle increased from ca. 700 nm at pH 7 to ca. 990 nm at pH 4 as a result of the repulsion between polymer chains. In alkaline media, HMSN-PDEAEMA was completely deprotonated to the hydrophobic polymer, reducing the particle size from 900 to 680 nm at pH 8.

The LC of HMSN-PDEAEMA and HMSN-PDEAEMA-glucosamine was determined by UV–vis spectroscopy at 480 nm using DOX as a guest molecule. At pH = 3, protonation of PDEAEMA leads to pore opening; thus, DOX can be hosted into the pores due to the electrostatic interaction between the drug and the internal surface of HMSNs. In contrast, at alkaline pH, the PDEAEMA shell collapses. The results indicate that the storage amount increases with DOX concentration. No significant difference in the LC was observed between HMSN-PDEAEMA and HMSN-PDEAEMA-glucosamine at the same concentration (see Table S1 and Figure S1).

**Figure 5:**
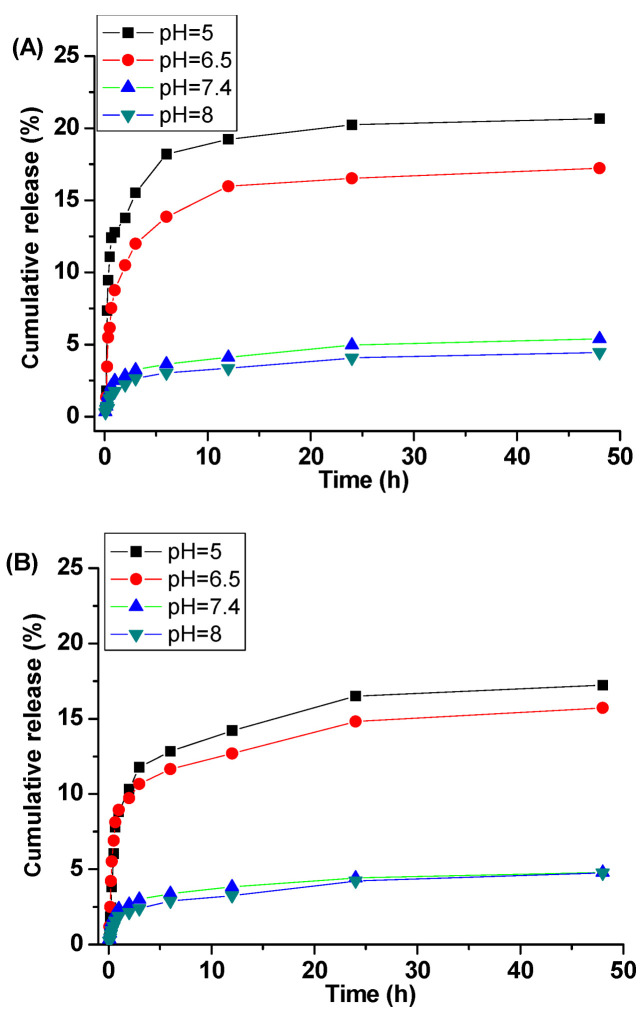
Drug release profiles of DOX loaded in: (**A**) HMSN-PDEAEMA and (**B**) HMSN-PDEAEMA-glucosamine nanocarriers in different pH buffer solutions at 37 °C.

The DOX release from HMSN-PDEAEMA and HMSN-PDEAEMA-glucosamine nanoparticles was studied in PBS buffer at pH 5, 6.5, 7.4, and 8. As illustrated in Figure 5, the drug release rate for both nanosystems was faster in mild acidic solution than in basic media. There was a slight difference in the cumulative drug release for both materials at pH 5 and 6.5, which was ca. 20% and ca. 18%, respectively, after 48 h. This is due to the fact that protons can easily reach the polymer chains and protonate the amine group of DOX, which accelerates the drug release. However, the amount of cumulative drug released from HMSN-PDEAEMA and HMSN-PDEAEMA-glucosamine decreased significantly at pH 7.4 and 8 to ca. 4% after 48 h. In these conditions, most of the drug was encapsulated in the nanocarrier due to the collapse of the polymer chains.

**Figure 6:**
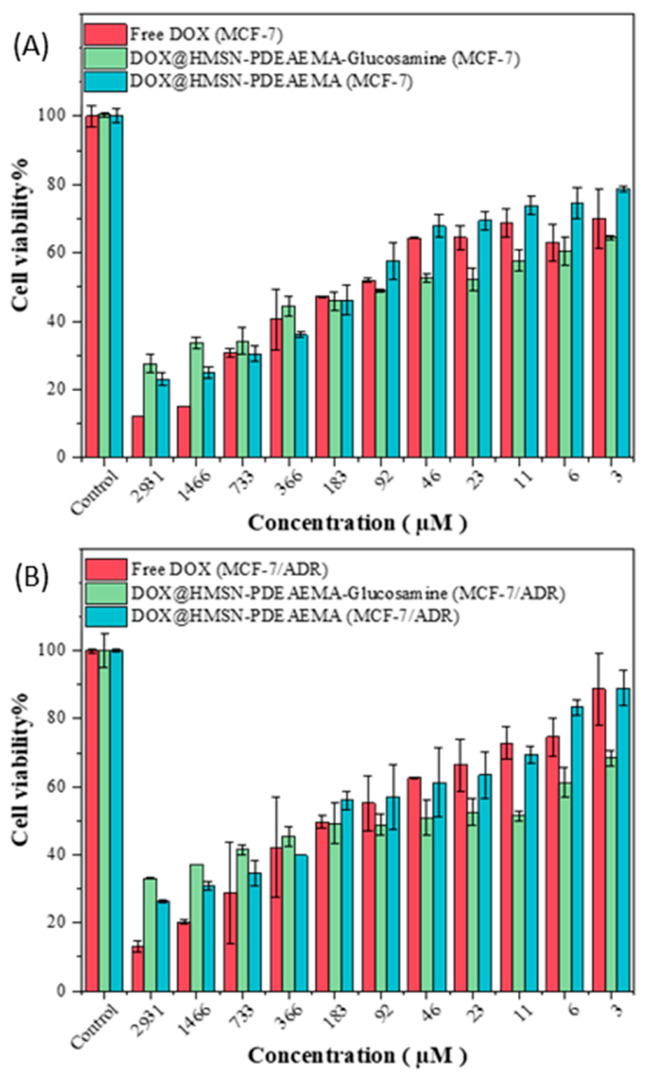
(**A**) The cell viability of MCF-7/ADR cells after incubation Free-DOX, DOX@HMSN-PDEAEMA and DOX@HMSN-PDEAEMA-Glucosamine. (**B**) The cell viability of MCF-7 cells after incubation Free-DOX, DOX@HMSN-PDEAEMA, and DOX@HMSN-PDEAEMA-Glucosamine.

The cytotoxicity effects of Free-DOX, DOX@HMSN-PDEAEMA, DOX@HMSN-PDEAEMA-Glucosamine, HMSN-PDEAEMA, and HMSN-PDEAEMA-Glucosamine were tested against the breast cancer cell line (MCF-7) and resistant breast cancer cell line toward Doxorubicin (MCF-7/ADR). Cancer cell lines kept high cell viability when exposed to HMSN-PDEAEMA and HMSN-PDEAEMA-Glucosamine for 24 h. (Figure S2). When cancer cell lines were incubated with all drug forms for 24 h to measure the cell toxicity, Free DOX demonstrated higher toxicity on both MCF-7 and MCF-7/ADR at only high drug concentrations (2931, 1465.5, 733, 366, and 183 µM). On the other hand, DOX@HMSN-PDEAEMA-Glucosamine showed strong killing ability to kill breast cancer cells in both types at low drug concentrations (23, 11, 6, and 3 µM) in comparison to the Free-DOX and the DOX@HMSN-PDEAEMA nanoparticles, *p* value (< 0.0001), as shown in Figure 6A,B.

## 4. Conclusions

In summary, we reported a facile synthesis of glucosamine-modified PDEAEMA brushes grafted on the outer surface of HMSNs via ATRP. The SiO_2_ core was coated with 50 nm mesoporous silica, followed by etching in alkaline media. The external surface of the nanoparticles was functionalized with an ATRP initiator, followed by growing PDEAEMA via ARGET ATRP method. The end-group functionality was converted to amine groups using sodium azide and triphenylphosphine. The amine groups were then reacted with succinic anhydride, followed by NHS activation, and glucosamine reaction. The fabricated nanomaterials were characterized by SEM, TEM, FTIR, TGA, and XPS. DLS was used to investigate the conformation change of the polymer chains at different pH values. The size of the nanoparticles increased twice in acidic media due to protonation of the tertiary amine in PDEAEMA, and the size decreased at pH above 7.4. DOX was efficiently loaded within the HMSN-PDEAEMA and HMSN-PDEAEMA-glucosamine nanocarriers, which exhibited an LC of ca. 64%. Furthermore, the release of DOX proceeded in a relatively controlled pH-triggered manner from both nanocarriers. The cytotoxicity studies demonstrated that DOX@HMSN-PDEAEMA-Glucosamine showed strong killing ability to kill breast cancer cells (MCF-7 and MCF-7/ADR) at low drug concentrations, in comparison to free DOX.

## Supplementary Materials

The following are available online at https://www.mdpi.com/2073-4360/12/11/2749/s1, Figure S1: Dox loading capacity of HMSNs-PDEAEMA and HMSNs- PDEAEMA- glucosamine, Figure S2: Illustration the effect of unloaded DOX hybrid nanoparticles on MCF-7 and MCF-7/ADR cells; Table S1: The loading capacity and the entrapment efficiency before and after glucose modification at different concentration of Dox. (a, c) Dox (0.5 mg/mL), (b, d) Dox (1 mg/mL).
