# Supplementary material for: Glucosamine Modified the Surface of pH-Responsive Poly(2-(diethylamino)ethyl Methacrylate) Brushes Grafted on Hollow Mesoporous Silica Nanoparticles as Smart Nanocarrier
Source: Polymers (Basel). 2020 Nov 20;12(11):2749. doi: 10.3390/polym12112749 (PMC7699838; doi:10.3390/polym12112749)
Supplement: Supplementary file 1 [file polymers-12-02749-s001.pdf]

Glucosamine modified the surface of pH-responsive poly(2-(diethylamino)ethyl methacrylate) brushes grafted on hollow mesoporous silica nanoparticles as smart nanocarrier

Abeer M. Beagan,[1*] Shatha S. Lahmadi,[1] Ahlam A. Alghamdi,[1] Majed A. Halwani,[2] Mohammed S. Almeataq,[3] Abdulaziz N. Alhazaa,[4] Khalid M. Alotaibi[1] and Abdullah M. Alswieleh[1*]

[1] Department of Chemistry, College of Science, King Saud University, Riyadh, Kingdom of Saudi Arabia.

[2] Nanomedicine Department, King Abdullah International Medical Research Center, King Saud bin Abdulaziz University for Health Sciences, Riyadh, Saudi.

[3] King Abdulaziz City for Science and Technology, Riyadh, Saudi Arabia.

[4] Department of Physics and Astronomy, College of Science, King Saud University, Riyadh, Saudi Arabia

* Corresponding Authors: A. Alswieleh, Email: aswieleh@ksu.edu.sa

A. Beagan, Email: abeagan@ksu.edu.sa

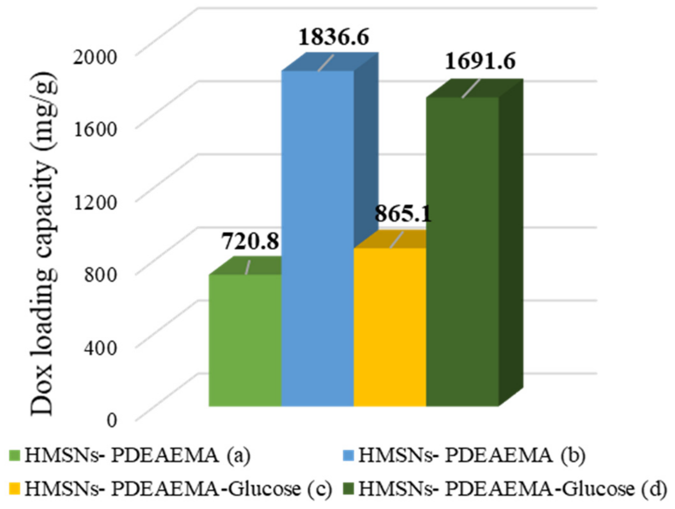

Figure S1: The dox loading capacity of HMSNs-PDEAEMA and HMSNs- PDEAEMA- glucosamine.

Table S1: The loading capacity and the entrapment efficiency before and after glucose modification at different concentrations of Dox. (a, c) Dox (0.5mg/ml), (b, d) Dox (1mg/ml).

Sample ID	*loading capacity%*	*Entrapment efficiency%*
HMSNs- PDEAEMA (a)	41.9%	72.1%
HMSNs- PDEAEMA (b)	64.7%	91.8%
HMSNs- PDEAEMA- glucose (c)	46.4%	86.5%
HMSNs- PDEAEMA- glucose (d)	62.8%	84.6%

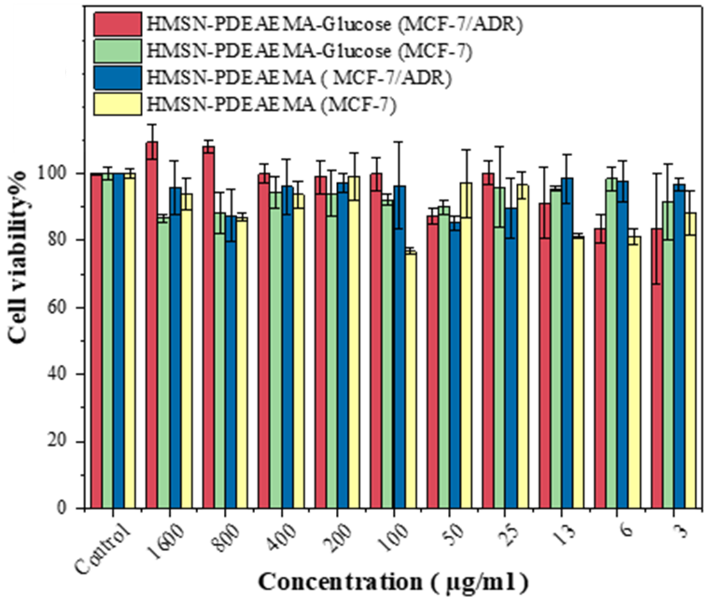

Figure S2. Illustration the effect of unloaded DOX hybrid nanoparticles on MCF-7 and MCF-7/ADR cells.
